# Six Common Mistakes in Conservation Priority Setting

**DOI:** 10.1111/cobi.12051

**Published:** 2013-04-08

**Authors:** Edward T Game, Peter Kareiva, Hugh P Possingham

**Affiliations:** * Conservation Science, The Nature Conservancy245 Riverside Drive, West End, QLD, 4101, Australia email egame@tnc.org; † Conservation Science, The Nature Conservancy4722 Latona Avenue NE, Seattle, WA, 98105, U.S.A.; ‡ Centre of Excellence for Environmental Decisions, University of QueenslandSt. Lucia, QLD, 4072, Australia

**Keywords:** Conservation Action Planning, conservation planning, decision science, measurement theory, operations research, prioritization, ciencia de la decisión, investigación de operaciones, Planificación de Acciones de Conservación, planificación de la conservación, priorización, teoría de medidas

## Abstract

**Abstract:**

A vast number of prioritization schemes have been developed to help conservation navigate tough decisions about the allocation of finite resources. However, the application of quantitative approaches to setting priorities in conservation frequently includes mistakes that can undermine their authors’ intention to be more rigorous and scientific in the way priorities are established and resources allocated. Drawing on well-established principles of decision science, we highlight 6 mistakes commonly associated with setting priorities for conservation: not acknowledging conservation plans are prioritizations; trying to solve an ill-defined problem; not prioritizing actions; arbitrariness; hidden value judgments; and not acknowledging risk of failure. We explain these mistakes and offer a path to help conservation planners avoid making the same mistakes in future prioritizations.

Seis Errores Comunes en la Definición de Prioridades de Conservación

**Resumen:**

Se ha desarrollado un vasto número de esquemas de priorización para ayudar a que la conservación navegue entre decisiones difíciles en cuanto a la asignación de recursos finitos. Sin embargo, la aplicación de métodos cuantitativos para la definición de prioridades en la conservación frecuentemente incluye errores que pueden socavar la intención de sus autores de ser más rigurosos y científicos en la manera en que se establecen las prioridades y se asignan los recursos. Con base en los bien establecidos principios de la ciencia de la decisión, resaltamos seis errores comúnmente asociados con la definición de prioridades para la conservación: no reconocer que los planes de conservación son priorizaciones; tratar de resolver un problema mal definido; no priorizar acciones; arbitrariedad; juicios de valor ocultos y no reconocer el riesgo de fracasar. Explicamos estos errores y ofrecemos un camino para que planificadores de la conservación no cometan los mismos errores en priorizaciones futuras.

## Introduction

Finite conservation resources and large environmental problems mean society is faced with tough choices; every good thing we do is another good thing we do not (Gilbert 2011). To help make these tough choices, many conservation–prioritization schemes have been developed (Kirkpatrick 1983; Brooks et al. 2006; Wilson et al. 2006). Prioritization schemes inform a wide array of conservation decisions, including prioritizing locations to establish protected areas, species to conserve, actions to conserve a species, and actions to address a particular threat. A quick glance at recent conservation text books or journal issues reveals that prioritization has become one of the principal pillars of conservation science. For example, a Web of Science search with the keywords “conservation” and “prioritization” contained 193 relevant publications between January 2010 and December 2011. It is important to appreciate there is a well-established scientific field that addresses the setting of priorities, that is decision science or operations research. These fields of science are a blend of applied mathematics, economics, philosophy, and psychology (Keeney 1982; Bell et al. 1988). Decision science aims to help people make the best decision in pursuit of a stated objective, particularly in situations that are highly complex or uncertain—common characteristics of conservation problems. Decision science together with operations research represents an extensive body of theory, methods, and tools that underpin much decision making in engineering, health, economics, and the military. Conservation is a relative newcomer to the field of decision science.

The burgeoning literature and application of quantitative priority setting in conservation demonstrates a desire for the field to become more rigorous and scientific. This is a good sign for conservation. Unfortunately, it is our experience that the application of quantitative approaches to setting priorities in conservation frequently includes mistakes that can undermine their authors’ intention to be more rigorous and scientific in the way priorities are established and resources allocated. Here, we highlight 6 critical mistakes associated with setting priorities for conservation. These are mistakes we have commonly seen both, in practice while working with NGOs and government agencies, and in the conservation–prioritization literature. Our goal is to explain these mistakes, without delving deeply into math or philosophy, and offer ways to avoid these mistakes in future prioritizations. Conservation has too few resources for its daunting challenges to be misapplying or not taking advantage of decision science.

### Mistake 1: Not Acknowledging Conservation Plans are Prioritizations

Scientists conducting conservation prioritizations (hereafter conservation planners) are not generally the same people making conservation decisions. The separation of these roles can lead to reluctance on behalf of conservation planners to explicitly frame analyses as resource prioritizations, preferring instead more neutral terms such as *conservation assessment* or *decision-support tool*. In our experience, this reluctance frequently stems from a belief (or knowledge) that decision makers will be unsupportive of plans that appear to reduce flexibility in their decision making or make potential actions harder to justify.

But what is a conservation plan if not a delineation of priorities? If the aim of analyses is to inform resource-allocation decisions at any level, it is a prioritization. Whether it is framed as prioritization, decision support, planning, analysis, or assessment, the principal function of these exercises is synthesizing and communicating information to help people reach better decisions. Although none of the terms above are inaccurate, we believe the widespread failure to either recognize or acknowledge conservation plans or assessments as resource prioritizations diminishes the utility of the work and precipitates a number of the mistakes highlighted below. The most immediate and significant consequence of this mistake is that analyses are not framed to answer a specific resource-allocation problem (mistake 2) and have outcomes that tend to ignore resource requirements and availability.

Avoiding this mistake requires acknowledging that conservation prioritizations are ultimately intended to have resource-allocation consequences, and by being explicit about what these resources and consequences are. The acceptability of any prioritization can be improved by ensuring that the motivations of decision makers, including political realities, are reflected in the problem framing (see mistake 2). Neither solution guarantees that prioritizations will be enthusiastically embraced by decision makers, but this should not stop conservation planners from providing the best advice possible.

### Mistake 2: Trying to Solve an Ill-Defined Problem

It is common in conservation to frame a priority-setting exercise with a statement about efficient use of resources—that is of course the goal of formal analyses aimed at setting priorities. However, too often in conservation these general statements are as far as problem framing goes. The most basic principles of decision science are that to set priorities one has to have a clear objective function (what is being maximized or minimized), have a well-defined set of actions from which a subset will be chosen as priorities, have a model of system behavior so that one can relate actions to their contributions toward meeting the objectives, and include resource constraints. Conservation problems are typically complex, and there will be varying emphasis on each of these elements. If they are not clearly defined, there is no basis for prioritization and there can be no claims of efficient resource use. In our collective experience, a poor definition of the problem or no definition of the problem is the most common mistake made in conservation priority setting.

Conservation planners often have trouble explicitly defining prioritization problems because the objectives relevant to the decision have not been clearly articulated. Many conservation initiatives seek to, for example, maintain ecosystem function, but this could mean simply maintaining function within a given area or it could imply using the area to maintain the function of a wider ecosystem. Precision is important because priorities are likely to be different depending on which of these 2 interpretations better reflects the actual desired outcome (e.g., Game et al. 2008a). Problem framing in conservation also suffers from poor articulation of the constraints on decisions, especially the extent to which prior decisions and existing commitments affect future actions. In fairness to conservation planners, there is often reluctance on the part of decision makers to explicitly acknowledge the preferences and constraints that affect their decisions. We believe decision makers should be encouraged to do so. As with mistake 1, we suspect the deeper cause of this mistake is that conservation planners are rarely the same people who allocate money or commit organizations to action; hence, they are operating in a decision vacuum.

The best way we can imagine overcoming obstacles to good problem framing is for planners to work in close partnership with decision makers from the very outset of any prioritization exercise. We recognize, however, that conservation plans are often undertaken by organizations or communities that are not in a position to partner with those responsible for making resource-allocation decisions, and yet they hope to influence these decisions. Even where this is the case, prioritization exercises can still be greatly strengthened by framing problems as realistically as possible and by ensuring the objectives included accurately reflect community and stakeholder values (Gregory 2000). Together, these 2 elements will make it more challenging for decision making to dismiss the resource-allocation consequences.

### Mistake 3: Not Prioritizing Actions

Conservation planners use prioritization where there are options in how resources might be used. Most conservation planners would be comfortable saying that they are prioritizing species, habitats, or locations. We argue that only actions can be legitimately prioritized. As outlined in mistake 1, prioritization is about resource-allocation decisions. Places, species, and habitats do not use the resources of conservation organizations and agencies—actions use resources. What a prioritization tells us is that some action associated with a location or species is a priority. Failing to acknowledge this from the outset is a recipe for inefficiency. Without being clear about actions, planners cannot confidently estimate how a given option might contribute to meeting objectives or the expected cost of the option, both of which are critical elements of any prioritization. Lists of priority species or locations without identification of associated actions are a good diagnostic symptom of this mistake.

Prioritizing actions does not mean ignoring places or species; it means understanding what one is going to do at those places or for those species, and prioritizing these combinations of places or species and action (e.g., Wilson et al. 2007; Joseph et al. 2009; Carwardine et al. 2012; Moore & Runge 2012). Quite often conservation planners prioritize actions without necessarily being aware of doing so. Consider, for example, prioritizations conducted by (or for) government agencies with the express purpose of identifying sites for new or expanded protected areas (e.g., Game et al. 2011). Protected-area establishment is an action; it has clearly identifiable costs and factors associated with it that are likely to promote or hinder it. However, at least an equal number of prioritizations are conducted without explicit statements of the actions associated with the priorities. The typical argument made in response to the suggestion that priorities need to reflect actions is that locations or species can be priorities because action needs to be taken for their conservation, but the best action cannot sensibly be determined a priori. Fair enough, but subsequent planning to determine the best action requires resources. Thus, determining the best action for these locations or species is an action in and of itself. Prioritizing locations or species for which to conduct further analyses and make subsequent decisions about resource allocation should consider things such as the actions that can be taken, how effective possible actions are, other resources being dedicated to finding solutions for that species, habitat, or location, and resources likely to be available for that location or species.

This mistake is commonly precipitated by committing mistake 1 or 2. If one has not thought clearly about the problem, there is a good chance one has not thought clearly about what it means to be a priority. There is also no doubt that understanding the set of available actions and their costs and benefits is more intensive and challenging than simply prioritizing a list of locations or species. Generation of such lists usually requires little critical thought. However, it is widely acknowledged in decision science that developing a good list of options from which to choose lies at the heart of good prioritization (Edwards 1990). Other than clearly defining the prioritization problem, the most effective remedy we have seen for this mistake is for conservation-planning exercises to dedicate time explicitly to canvasing experts, stakeholders, and decisions makers about potential actions that could be taken. A good check to see how well this has been accomplished is to ask, if a species, habitat, or location is selected as a priority, would it be clear what actions to take?

### Mistake 4: Arbitrariness

Conservation planners must routinely conduct prioritizations with less data than they would like and often without direct data on the variables they are most concerned with, such as habitat condition. Because simple surrogate measures do not always exist (or are not viewed as satisfactory), conservation prioritizations typically integrate data on a number of variables from a variety of sources, including in many cases much expert judgement. A common approach in such circumstances is to classify variables of interest, such as the level of threat to a species or habitat, with a constructed scale. For example, in assessing conservation priority of different habitat patches a conservation planner might score the disturbance to areas on a scale of 1–7. Constructed scales can even be based on simple linguistic interpretations (e.g., threat classified as high, medium, or low), which are then subsequently converted to numerical values (e.g., high, 3; medium, 2; low, 1).

The scores assigned to these variables are essentially arbitrary—there is no objective reason why a relatively undisturbed habitat should be given a score of 4 rather than 5 for example. What these constructed scales typically represent is a set of ordinal numbers that indicate, for example, a score of 2 is better than a score of 1 and worse than a score of 3. If one restricts interpretation of such scales to simple ordinal representations between alternatives (e.g., alternative *X* is better than alternative *Y* for variable *Z*), then the arbitrary nature of the numbers is not problematic. However, ordinal numbers do not convey how much better 2 is than 1; thus, constructed ordinal scales are a problem when one treats them as a set of regular numbers to be used in prioritization arithmetic (e.g., adding 2 or more variables together). For instance, to help rank the conservation priority of different habitats, The Nature Conservancy's Conservation Action Planning (CAP) process (and the conservation-planning software *Miradi*) combines measures of their size, condition, and landscape context with the following scale: very good, 4; good, 3.5; fair, 2.5; poor, 1. The overall rank is the arithmetic mean of these 3 categories. Consider 2 habitats, A and B. Habitat A receives 3 scores of fair, whereas habitat B receives 2 scores of good and one of poor. On the basis of the arithmetic mean, habitat B (8) ranks above habitat A (7.5). If one adjusted the choice of scale such that good was worth 3 rather than 3.5, habitat A (7.5) ranks above habitat B (7). As Wolman (2006) eloquently puts it, the “truth or falsity of results derived from measurements should not depend on a fortuitous choice of scale.”

An easy way to check whether a prioritization result is likely to be meaningful and not arbitrary is to go back to the underlying data. In the example above, a habitat rated very good (score of 4) must be unambiguously considered 4 times better than a habitat rated poor (1) because this is how it is being treated when the arithmetic mean is calculated. This mistake is best avoided by estimating variables of interest on natural scales wherever possible.

### Mistake 5: Hidden Value Judgements

Many conservation planners have an intuitive sense that variables do not affect priority equally, linearly, or independently. A common response to this realization is to establish a set of rules for combining variables, often presented in a look-up table. Look-up tables are alluring because they are an easy way to combine variables and because they can be developed by personnel without formal training in modeling. However, this ease of creation can lead to error.

Rules and look-up tables reflect the values, beliefs, assumptions, biases, and risk tolerances of their creators. For example, Table 1 (taken from a prioritization process 2 of the authors were subsequently involved with) shows how assessments of the size and context of habitat patches should be combined to determine overall priority rank. In this case, a fair for size and a fair for context result in an overall score of poor. Logically, one might expect this to yield an overall score of fair, but the rules in the table could reflect the planner's belief that there is an interaction between these variables that further reduces the conservation importance of the habitat patch at low scores or, alternatively, the planner's assessment that addressing a fair score for either size or context of a patch might be possible but addressing both factors would be unrealistic. One cannot be sure. Similarly, a poor for size and a very good for context results in an overall score of good, whereas a poor for context and a very good for size results in an overall score of fair. Again, one cannot be sure whether this means the conservation planner believes context should have more effect on priority than size, addressing context is more feasible than addressing size, or why this assessment of effect is limited to this combination of scores. Interpreting these values and judgements becomes a daunting prospect when the look-up tables contain 3 or more variables.

**Table 1:** Example of a look-up table illustrating how assessments of the size and context of habitat patches should be combined to determine an overall priority rank (taken from a prioritization process 2 of the authors were involved with)

	Size			
	
Context	very good	good	fair	poor
Very good	VG	VG	G	G
Good	VG	G	F	F
Fair	G	F	P	P
Poor	F	F	P	P

Abbreviations: VG, very good; G, good; F, fair; P, poor.

The principal issue here is not the questionable math (discussed in mistake 4), but that these judgements are not transparent and therefore not open to critique. Quantitative prioritizations are intended to reduce bias and promote objectivity or at least to be explicit about assumptions, bias, and their effects so that assumptions and the resulting priorities can be effectively contested by interested parties. Many involved in decision-making philosophy consider contestability the formative property of a defensible prioritization process (Burgman 2005). Rather than promoting transparency, planning methods that include, for example, look-up tables and combinatorial rules actually obfuscate the reasons behind the prioritizations by burying a series of value judgements and assumptions beneath a numerical veneer. Such a use of numbers simply formalizes unacknowledged bias and endows the process with a false credibility. Instead of using a flawed prioritization process, it would be better to acknowledge that priorities are based on individual intuition, bias and all. Donors and the public can then judge whether they are comfortable for their resources to be prioritized this way. Our experience has been that when value judgements and intuition are made transparent, they are likely to be challenged.

A prioritization conducted by The Nature Conservancy to help make decisions about establishing new conservation projects in Africa provides a good illustration of how this mistake can be addressed. Staff involved in the plan believed that, from The Nature Conservancy's perspective, the relative conservation priority of each country was affected by the distinctiveness of the biological diversity, extent of land clearing, level of fragmentation of the remaining habitat, extent of the existing protected-area network, and quality of governance in the country. To make value judgements about these variables transparent, one of us (E.T.G.) asked employees involved to sketch functions that reflected their belief about how each variable related to conservation priority. Having a strong preference for conserving less-fragmented habitat, for example, is perfectly legitimate, but this preference should be clearly distinguished from a scientific assessment of the effects of habitat fragmentation on conservation outcomes (Failing & Gregory 2003; Wilhere et al. 2012). These sketched functions were then turned into mathematical expressions and used as part of a return-on-investment prioritization process. Although drawing heavily on the experience, opinions, and values of the employees involved, the prioritization did so in an explicit fashion that made it possible to identify and contest these beliefs.

### Mistake 6: Not Acknowledging Risk of Failure

Nearly all conservation actions have a chance of failure. For example, if a conservation action involves eradicating a pest, this action often fails (technical failure). Similarly, some actions may fail because the people implementing the action fail (poor management) or because sociopolitical forces thwart the action (e.g., loss of community support, change of government interest). Failure due to poor management can generally be mitigated, but stochastic events that lead to project failure are unavoidable (e.g., Game et al. 2008b). The probability that a conservation action will fail substantially affects the expected costs and benefits of that action, and yet this risk of failure is more often than not completely absent from conservation prioritizations. Although some assessment of risk may be implicit in conservation plans that rate or score options on the basis of feasibility or ease of implementation, these scores are typically added to scores for other variables to determine priority. We know of no situation where this is logical.

There are at least 2 contributing factors to the absence or poor treatment of risk in conservation prioritization. First, there is often strong reticence to acknowledge that conservation actions might fail, especially where the prioritization has a partial advocacy role or those involved have an interest in the implementation of a particular action (Redford & Taber 2000). Second, psychologists have repeatedly demonstrated that most people find it very challenging to rationally incorporate risks and probabilities into their judgements (Plous 1993). In prioritization the simplest and most logical way of acknowledging risk and its effects on the likelihood of conservation success is almost always through expressing these as probabilities (e.g., Game et al. 2008b; Joseph et al. 2009).

### A Path Forward

People use quantitative prioritization approaches in conservation because they want to do things better. There is logic behind most of the prioritization systems used in conservation; generally speaking they are moving in the right direction. However, by committing one or more of the mistakes described above, a large number of priority-setting exercises violate key principles of good, defensible decision support. As well as leading to poor resource allocation decisions, prioritizations that contain these mistakes can obfuscate the reasons behind decisions and give the prioritization a false credibility. This point is worth belaboring because priority-setting exercises are commonly presented as the principle science behind conservation decisions. Public confidence requires credibility (Wilhere et al. 2012). Objective treatment of empirical phenomena is a scientist's stock-in-trade and the source of scientists’ authority. It is perfectly legitimate for opinions and value judgements to affect resource- allocation decisions—especially in a field such as conservation—but they should be clearly distinguished from scientific evidence.

The field of decision science has provided information and tools to ensure that prioritizations deliver objective, defensible, and ultimately efficient conservation decisions (e.g., Keeney & Raiffa 1993; Gregory et al. 2012). The mistakes highlighted here reflect either an absence of understanding or commitment to decision science. In addition to the specific antidotes we mentioned with each mistake, we believe the quality and usefulness of conservation priority setting can be improved by broader recognition that conservation planners act as both modelers and decision analysts and need to be trained in the science and philosophy of these disciplines. We hope that highlighting these common mistakes in conservation priority setting will encourage conservation planners to learn more about decision science and the principles that underpin their own work.
